# Lowering of the mouth floor and vestibuloplasty to support a mandibular overdenture retained by two implants. A case report

**DOI:** 10.4317/jced.51402

**Published:** 2014-07-01

**Authors:** Isidoro Cortell-Ballester, Rui Figueiredo, Cosme Gay-Escoda

**Affiliations:** 1 DDS. Master in Oral Surgery and Orofacial Implantology, School of Dentistry, University of Barcelona, Spain; 2 DDS. Associate Professor of Oral Surgery. Professor of the Master degree program in Oral Surgery and Orofacial Implantology. School of Dentistry of the University of Barcelona. Researcher of the IDIBELL Institute. Barcelona, Spain; 3 DDS, MD, PhD. Chairman and Professor of Oral and Maxillofacial Surgery. School of Dentistry of the University of Barcelona. Coordinator/Researcher of the IDIBELL Institute. Oral and Maxillofacial Surgeon of the Teknon Medical Center, Barcelona, Spain

## Abstract

In Oral Implantology most of the procedures are predictable and have high success rates. The use of osseointegrated implants as a therapeutic option for the rehabilitation of patients with severe mandibular atrophy has decreased the need for pre-prosthetic surgery Nevertheless, complications may occur during implant surgery and also once the prosthesis has been placed. This paper describes the case of a totally edentulous patient with an upper complete removable denture and an implant-retained overdenture with two implants in the intermentonian region. During clinical examination, the implant abutments were totally covered by soft tissue since the floor of the mouth was elevated. The panoramic radiography showed severe mandibular atrophy. Vestibuloplasty was performed together with the lowering of the floor of the mouth under general anesthesia and nasotracheal intubation to expose the implants. A new prosthesis was fabricated for the patient to prevent recurrence and improve the patient’s chewing ability as it formed a physical barrier against soft tissue migration on prosthetic attachments.

** Key words:**Vestibuloplasty, lowering of the mouth floor, complications in oral implantology.

## Introduction

The rehabilitation of resorbed edentulous mandibles can be a challenge for a variety of reasons. These may include a lack of bone, exostosis, tori, genial tubercle obstruction or an elevated floor mouth (1).

The use of osseointegrated implants as a therapeutic option for the rehabilitation of patients with severe mandibular atrophy has decreased the need for pre-prosthetic surgery (2). Nevertheless, such techniques should be taken into account when modification of soft tissues is required [lack of keratinized gingiva, soft tissue enlargement, decreased vestibular depth and elevated floor mouth] since they enhance prosthesis retention and create an adequate placement area, even when dental implants are going to be used.

In this paper, we present the case of a woman with an upper complete removable denture and an implant-retained mandibular overdenture with 2 implants that required a vestibuloplasty together with the lowering of the floor to expose the prosthetics abutments.

## Case Report

**Figure 1:**
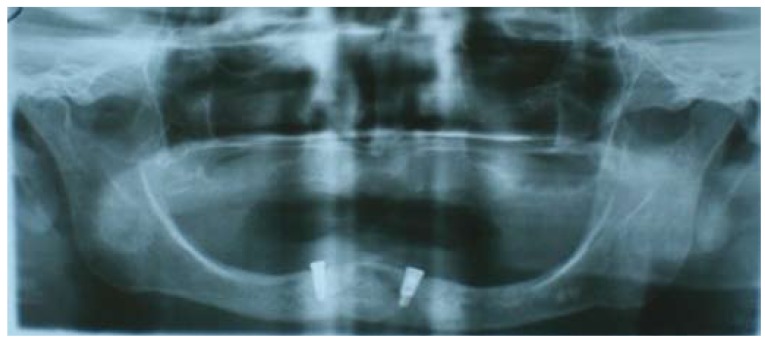
Orthopantomogram. Severe mandibular atrophy and 2 osseointegrated implants in the intermentonian region.

A 75-year old Caucasian female with insulin-dependent diabetes mellitus. The patient was totally edentulous with an upper complete removable denture and an implant-retained overdenture with 2 implants in the intermentonian region. When she first visited the Service of Oral Implantology of the Dental Clinic of the University of Barcelona, the prosthetic abutments [ball-shaped anchors] were completely covered by soft tissue. The clinical examination revealed an elevated floor of the mouth and a reduced vestibular depth. The panoramic radiography showed severe mandibular atrophy (Fig. 1).

**Figure 2:**
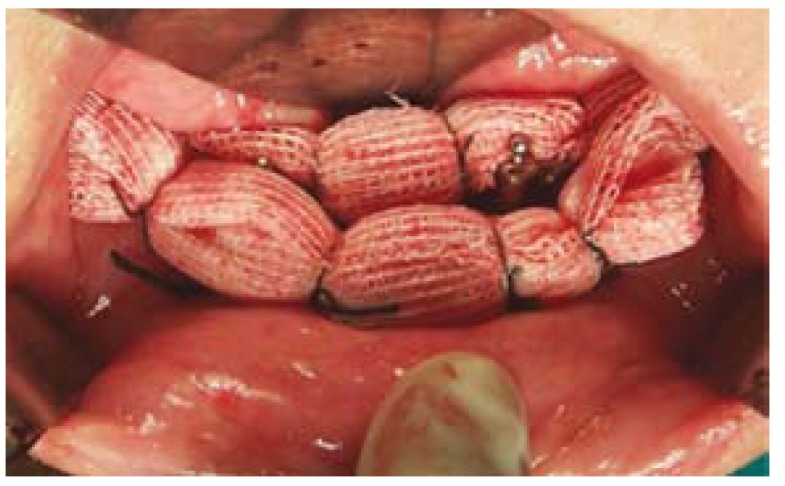
Immediate postoperative period. The two prothesic abutments where exposed with Linitul® dressings placed at the buccal and lingual sides in order to promote secondary healing.

**Figure 3:**
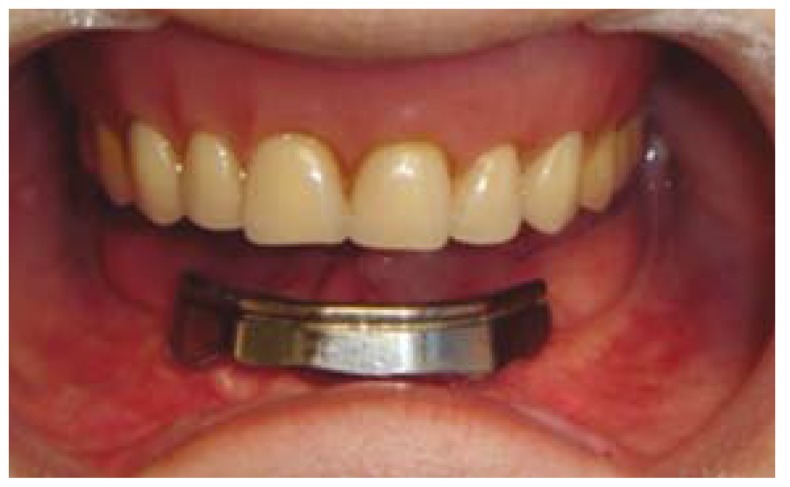
Createch Medical® bar for overdenture retention.

The vestibuloplasty and the lowering of the floor of the mouth were performed under general anaesthesia and nasotracheal intubation. A crestal incision was used and 2 partial-thickness flaps were raised, one buccal and one lingual, leaving the periosteum intact and adherent to the mandibular bone. Soft tissue dissection was carefully carried out in order to avoid mental nerve injuries. Once implant abutments were exposed, the flaps were repositioned through the placement of a transfixion suture surrounding the mandible. The soft tissues around the implants were left to heal by secondary intention and two wound dressings impregnated with castor oil and Balsam of Peru were placed in both buccal and lingual sides [Linitul®, Bama-Geve, Barcelona, Spain] (Fig. 2). After the operation, an antibiotic [amoxicillin 750 mg every 8 hours for 4 to 7 days [Clamoxyl 750; GlaxoSmithKline, Madrid, Spain], a nonsteroidal anti-inflammatory drug [sodium diclofenac 50 mg every 8 hours [Diclofenaco Llorens 50 mg; Llorens, Barcelona, Spain] or ibuprofen 600 mg every 8 hours for 4 to 5 days [Algiasdin 600; Esteve; Barcelona, Spain], an analgesic [metamizol 575 mg every 4 hours for 3 to 4 days [Nolotil; Boehringer Ingelheim, Sant Cugat del Vallès, Spain], and a mouthrinse [0.12% chlorhexidine digluconate every 12 hours for 15 days [Clorhexidina Lacer; Lacer, Barcelona, Spain] were prescribed together with the placement of ice packs. Postoperative instructions and prescribed drugs were explained and printed on a paper that was given to the patient. The dressings were removed one week after surgery and the transfixion sutures were removed after 15 days. At one month, the soft tissues surrounding the prosthetic abutments were totally healed. A new prosthesis was then made. In this case a Createch Medical® bar [Createch Medical®, Mendaro, Guipúzcua, Spain] was placed to prevent the soft tissue to cover the prosthetic abutments (Fig. 3).

## Discussion

Most of the dental implant rehabilitations are predictable and have high success rates. However surgical complications can arise following such treatments (2). The use of osseointegrated implants has led to a decreased need for more traumatic surgical procedures, such as vestibuloplasties [with or without skin grafting] and lowering of the mouth floor, that were originally conceived and designed when the only therapeutic option was the use of complete removable dentures. However, such techniques may be indicated in specific cases where soft tissue modification is required for oral rehabilitation with osseointegrated implants and when implant-retained or implant-supported prostheses are a viable treatment option. Areas of soft tissue enlargement, lack of keratinized gingiva, decreased vestibular depth or elevated mouth floor are conditions that can affect prosthetic stability, retention and function; and therefore, may need surgery to correct these problems.

The functional reconstruction of the edentulous mandible with implant-supported overdentures has become an accepted treatment option for several authors (1,3,4). Some studies have reported significant improvement in chewing ability and efficiency after treatment with this type of prosthesis used in patients with severe maxillary resorption as well as improved patient’s satisfaction (3,4).

Bone resorption and the use of removable prostheses negatively affect quantity and quality of keratinized mucosa. Furthermore, these patients usually present high muscle insertions, which leads to abnormal tongue position or function with a subsequent lack of stability and unsatisfactory prosthetic function. In this context, the lowering of the mouth floor maybe is recommended prior to implant surgery (5).

Most authors agree that vestibuloplasties should be performed 2 months before implant placement. However the simultaneous placement of implants has reported good results in terms of prosthetic function and patient satisfaction (1,5-7).

Cillo and Finn (1) evaluated the implant survival and prosthetic success in 13 patients [40 implants] who underwent simultaneous mandibular vestibuloplasty with partial thickness skin graft and endosseous implant placement for implant-supported overdentures. The implant survival rate was 97.5% after 43 months of follow-up. There were no complications in graft or recipient sites. All patients had satisfactory retention, stability, and function of their implant-supported mandibular overdenture. Furthermore, Kao *et al*. (7) reviewed the use of a transpositioned flap vestibuloplasty combined with implant surgery in 17 patients with severely resorbed atrophic edentulous ridges. After a follow-up period of 6 years, satisfactory results were observed regarding the stability of implant fixtures and the functionality of the prostheses. In our case, a vestibuloplasty either before or during simultaneous surgery for implant placement would have avoided the need for 2 overdentures and would have therefore reduced patient discomfort and pain. Nevertheless, the vestibuloplasty and the lowering of the floor of the mouth allowed for exposure of the attachments placed onto the implants with better soft tissue adaptation. Furthermore, the design of the new prosthesis fabricated for the patient also prevented recurrence as it formed a physical barrier against soft tissue migration on prosthetic attachments.
